# A Novel *FC116*/*BC10* Mutation Distinctively Causes Alteration in the Expression of the Genes for Cell Wall Polymer Synthesis in Rice

**DOI:** 10.3389/fpls.2016.01366

**Published:** 2016-09-21

**Authors:** Mingliang Zhang, Feng Wei, Kai Guo, Zhen Hu, Yuyang Li, Guosheng Xie, Yanting Wang, Xiwen Cai, Liangcai Peng, Lingqiang Wang

**Affiliations:** ^1^Biomass and Bioenergy Research Centre, Huazhong Agricultural UniversityWuhan, China; ^2^National Key Laboratory of Crop Genetic Improvement, Huazhong Agricultural UniversityWuhan, China; ^3^College of Plant Science and Technology, Huazhong Agricultural UniversityWuhan, China; ^4^College of Life Science and Technology, Huazhong Agricultural UniversityWuhan, China; ^5^Department of Plant Science, North Dakota State UniversityFargo, ND, USA

**Keywords:** rice, cell wall, cellulose, fragile culm, mechanical strength

## Abstract

We report isolation and characterization of a fragile culm mutant *fc116* that displays reduced mechanical strength caused by decreased cellulose content and altered cell wall structure in rice. Map-based cloning revealed that *fc116* was a base substitution mutant (G to A) in a putative beta-1,6-N-acetylglucosaminyltransferase (C2GnT) gene (LOC_Os05g07790, allelic to *BC10*). This mutation resulted in one amino acid missing within a newly-identified protein motif “R, RXG, RA.” The *FC116/BC10* gene was lowly but ubiquitously expressed in the all tissues examined across the whole life cycle of rice, and slightly down-regulated during secondary growth. This mutant also exhibited a significant increase in the content of hemicelluloses and lignins, as well as the content of pentoses (xylose and arabinose). But the content of hexoses (glucose, mannose, and galactose) was decreased in both cellulosic and non-cellulosic (pectins and hemicelluloses) fractions of the mutant. Transcriptomic analysis indicated that the typical genes in the *fc116* mutant were up-regulated corresponding to xylan biosynthesis, as well as lignin biosynthesis including *p*-hydroxyphenyl (H), syringyl (S), and guaiacyl (G). Our results indicate that FC116 has universal function in regulation of the cell wall polymers in rice.

## Introduction

Plant cell walls are complex, dynamic cellular structures essential for plant growth, development, physiology, and adaptation. Cell walls can be classed as primary or secondary, depending upon whether they are extendable or non-extendable during organ growth (Lee et al., 2011). Primary cell wall is a flexible matrix that mainly composed of cellulose, hemicelluloses, pectins, and proteins, allowing directed cell growth. Specialized cell types differentiate by depositing a secondary cell wall, providing mechanical support for water and nutrient transport as well as the barrier against invading pathogens (Cantu et al., 2008; Wang and Dixon, 2012). In general, cellulose makes up almost 25–30% of dry matter in grasses (Zhang et al., 2013) and 40–45% in woody plants (Smook, 1992). Hemicelluloses include polysaccharides such as xyloglucans, xylans, mannans, and glucomannans, and β-(1,3-1,4)-glucans (Scheller and Ulvskov, 2010). Lignin is an amorphous polymer of phenylpropane units with three major monomers: *p*-hydroxyphenyl (H), guaiacyl (G), and syringyl (S), laid down during secondary cell wall formation (Zhao and Dixon, 2011; Sun et al., 2012).

The polymers contained within the cell wall are an important renewable resource for humans as dietary fiber, as raw material for pulp manufacturing, and for biofuel production (Taylor-Teeples et al., 2015). Elucidation of the regulatory mechanisms of cell wall synthesis will facilitate the engineering of plant feedstocks suitable for biofuel production (Wang and Dixon, 2012). Although significant progress has been made in understanding the regulation of secondary cell wall formation in *Arabidopsis thaliana* (Zhong and Ye, 2007; Demura and Ye, 2010; Wang and Dixon, 2012; Taylor-Teeples et al., 2015), our knowledge of the precise regulatory mechanisms of cell wall biosynthesis is still limited especially in monocot plants. Thousands of genes have been reported to be responsible for plant cell wall biogenesis and modifications (Carpita et al., 2001; Torney et al., 2007; Xie and Peng, 2011). Among the products of the genes, glycosyltransferases (GTs) are one of the largest enzyme groups required for the synthesis of complex cell wall polysaccharides and glycoproteins in plants (http://www.cazy.org; Rosén et al., 2004; Scheible and Pauly, 2004). Further, the rapid increase in the availability of genomic resources and improvement in molecular techniques have led to additional GTs being identified through bioinformatic analysis. However, only a small portion of the genes involving in cell wall formation have been functionally characterized primarily in model organisms *A. thaliana* and rice (Somerville et al., 2004; Zhang and Zhou, 2011).

A powerful approach to probe the functions of individual components of cell walls is through the identification and characterization of relevant mutants (Reiter et al., 1993). Over the last two decades, dozens of genes have been identified to be involved in cell wall synthesis and modifications by characterizing cell wall mutants in Arabidopsis and rice (Turner and Somerville, 1997; Sun et al., 2012). Studies on the brittle culm (*bc*) mutants of rice have identified several genes responsible for secondary cell wall biosynthesis. These studies have revealed some mechanistic aspects of cell wall biosynthesis and identified associated genes in rice (Zhang and Zhou, 2011). *BC7*(*t*) and *BC11* encoding cellulose synthases are assembled into the cellulose synthase complex (CSC) in Golgi and then translocated to the plasma membrane (Yan et al., 2007). *BC1* encodes a COBRA-like protein that is released to the extracellular matrix and is essential for cellulose assembly (Li et al., 2003; Sato et al., 2010). *BC3* encoding dynamin-related proteins (DRPs) functions in membrane vesiculation and trafficking (Hirano et al., 2010; Xiong et al., 2010). *BC12/GDD1* encodes a kinesin-like protein secreted to the cytoplasm and functions in the cortical microtubule-dependent cellulose deposition and in regulation of GA biosynthesis (Zhang et al., 2010; Li et al., 2011). *BC15* encodes a membrane-associated chitinase-like protein, a class of glycosyl hydrolases that are required for cellulose biosynthesis (Wu et al., 2012). More recently, OsMYB103L, an R2R3-MYB transcription factor, was found involved in GA-mediated regulation of secondary cell wall biosynthesis. It influences leaf rolling and mechanical strength in rice (Yang et al., 2014; Ye et al., 2015).

Among the brittle culm genes, *BC10* is proposed to encode a protein that belongs to a newly-identified glycosyltransferase family in plants. It is located in Golgi or part of the structural and is regulatory protein for cell wall remodeling/formation (Zhou et al., 2009). Mutation in the “domain of unknown function 266” (DUF266) of BC10 resulted in a truncated protein that modifies the levels of cellulose and arabinogalactan proteins (AGPs) in cell walls and leads to brittleness of the plant parts and retarded growth. Since DUF266 is the only domain predicted using Pfam and is plant-specific in this protein family, it may be critical for the function of BC10 (Finn et al., 2006). It was predicted that the mutation in *bc10* probably leads to perturbation of the biosynthesis of glycoprotein and/or non-cellulosic polysaccharides (Zhou et al., 2009). However, the precise functions of BC10 proteins and their roles in cellulose biosynthesis and secondary cell wall formation have not been fully elucidated. How the *bc10* mutation influences biosynthesis of cellulose and AGP as well as other cell wall polymers remains elusive. Here, we reported the characterization and map-based gene cloning of a novel rice fragile culm mutant designated *fc116* that exhibits abnormal sclerenchyma secondary cell walls in the internodes.

## Materials and methods

### Plant materials and growth conditions

The fragile culm mutant *fc116* was isolated from the EMS-induced mutant pool of the *japonica* rice cultivar “Zhonghua 11” (ZH11) in year 2010, and was crossed with two different rice cultivars “Minghui 63” (MH63) and “9311,” respectively. Two F_2_ mapping populations were generated and planted in the field in year 2013 and map-based cloning was conducted to identify the mutant allele responsible for the *fc116* phenotype. The field experiments were conducted in the rice-growing seasons on the experimental farm of Huazhong Agricultural University, Wuhan, China. The sowing dates were May 25. Ten plants per line were transplanted in a single row with 16.5 cm between plants and 26.4 cm between rows. Field management essentially followed normal agricultural practice.

### Measurement of plant mechanical properties

The second internodes of stem and flag leaves of wild-type and *fc116* plants were tested for mechanical properties at the milk maturity stage. The stems and leaves were cut into segments of 5 cm in length. The stretching force of the samples before being broken was measured with a universal force/length testing device (model RH-K300, Guangzhou, China). The newton is used as the unit of extension force (EF). The stem tissues at 30 days after flowering were used to detect the plant lodging index. The breaking resistance of the third internode was detected using a Prostrate Tester (DIK 7401, Japan), with the distance between fulcra of the tester at 5 cm. The newton is used as the unit of breaking force (BF). Fresh weight (W) of the upper portion of the plant was measured including panicle and the three internodes, leaf, and leaf sheath. Bending moment (BM) and lodging index (LI) were calculated using the following formula: BM = Length from the third internode to the top of panicle × W, LI = BM/BF

### Map-based cloning of *FC116* gene

The initial mapping of *fc116* was performed using 289 SSR markers distributed throughout the rice genome in bulked pools and parents. The genomic region harboring the *fc116* locus was saturated with newly developed molecular markers. Using 1917 F_2_ plants derived from the cross of the mutant *fc116* with MH63, the mutated locus was finally narrowed down to a 202-kb region on chromosome 5. The corresponding DNA and cDNA fragments were amplified from *fc116* and its wild type “Zhonghua 11” (ZH11) plants using KOD plus (Toyobo, www.toyobo-global.com) and sequenced with a 3730 sequencer (ABI).

### Bioinformatic analysis of FC116 protein

Prediction of FC116 protein domain was performed by the SMART (http://smart.embl-heidelberg.de/). The protein transmembrane helices were predicted by TMHMM2.0 (http://www.cbs.dtu.dk/services/TMHMM-2.0/). Three dimensional structure of FC116 was performed using SWISS-MODEL online prediction software (http://swissmodel.expasy.org/). The unrooted phylogenetic trees were constructed with the MEGA6 program and the neighbor joining method with 1000 bootstrap replicates. We performed exon-intron structure analysis using GSDS (http://gsds.cbi. pku.edu.cn/). FC116 protein sequences alignment was performed by ESPript 3.0 (http://espript.ibcp.fr/ESPript/cgi-bin/ESPript.cgi).

### Genome-wide expression analysis of *OSFC116* gene family

The expression profile data of *OsFC116* in 33 tissue samples (Table S2) of Zhenshan 97 (ZS97) and Minghui 63 (MH63) were obtained from the CREP database (http://crep.ncpgr.cn). The expression values were log-transformed, and cluster analyses were performed using a software cluster with Euclidean distances and the hierarchical cluster method of “complete linkage clustering.”

### Histochemical staining

For histochemical localization of lignin, a Wiesner reaction was performed according to Strivastava (1966) with minor modifications. Fresh hand-cut sections from the second culms were incubated for 1 min in phloroglucin solution [5% in ethanol: water (95:5, v/v)], mounted on slides with 37% HCl, and photographed using a microscope (Olympus BX61, Japan). For cellulose staining, fresh hand-cut sections were stained with 0.1% calcofluor (Fluka) for 2 min. Excess calcofluor was washed away with tap water and slides were visualized with a fluorescent microscope (Olympus BX61, Japan).

### Immunofluorescence detection

The sample preparation and immunohistochemical localization of xylan were performed as previously described by Cao (Cao et al., 2014). The main vein of leaves at the trefoil stage were cut into 0.2–1.5 cm pieces and subsequently fixed with glutaraldehyde. The above pieces were embedded by paraffin and sectioned into slices with 8.0 μm in thickness. Monoclonal antibody CCRC-M148 was used to detect xylan. The sections were incubated in PBS (phosphate buffer saline) contained 3% SMP (3% skim milk powder, w/v) containing 10 μg/ml monoclonal antibody CCRC-M148 for 2 h. The immunolabeled samples were washed three times (5 min each) with PBS and incubated with a 100-fold dilution of anti-mouse-IgG for the CCRC-M148 antibody in dark for 1 h. The anti-mouse-IgG antibody was labeled by fluorescein-isothiocyanate (FITC). Immunofluorescence sections were observed under an Olympus BX-61 microscope.

### Collection and preparation of cell wall fractions

The method of plant cell wall fractionation was used to extract cellulose and hemicellulose, as described by Peng et al. (2000) and Li et al. (2015). We used potassium phosphate buffer (pH 7.0), chloroform-methanol (1: 1, v/v), and DMSO-water (9: 1, v/v) to remove soluble sugar, lipids, and starch from the samples. The remaining pellets as total crude cell wells were suspended in 0.5% (w/v) ammonium oxalate and heated for 1 h in a boiling water bath, and the supernatants were total pectins. The remaining pellets were suspended in 4 M KOH containing 1.0 mg/mL sodium borohydride for 1 h at 25°C. The mixtures were then centrifuged and the supernatants were collected and regarded as KOH- extractable hemicelluloses. The remaining pellets were reacted with 2 M trifluoroacetic acid (TFA) and the supernatants were collected and regarded as non-KOH- extractable hemicelluloses. The pellets were further extracted with acetic-nitric acids-water (8: 1: 2) for 1 h at 100°C, and the remaining materials were regarded as crystalline cellulose. The supernatants extracted from 4 M KOH treatment and from TFA reaction were separately transfered into 5 mL screw-cap test tubes and were neutralized, dialysed, and lyophilized according to the method described by Xu et al. (2012), then subjected to GC-MS measurements or stored −20°C until GC-MS measurements. During the treatments, the screw-cap test tubes were sealed with adhesive tape to avoid possible sample lost.

### Colorimetric assay of hexoses and pentoses

The hexose and pentose assays were performed using an UV-vis spectrometer according to Li et al. (2013) (V-1100D, Shanghai MAPADA Instruments Co., Ltd. Shanghai, China). Hexoses were measured by the anthrone/H_2_SO_4_ method and pentoses by orcinol/HCl (Dische, 1962). As the high pentose level affects the absorbance reading at 620 nm for the hexose assay by the anthrone/H_2_SO4 method, the deduction from pentoses was carried out for a final hexose calculation. A series of xylose concentrations were analyzed for plotting the standard curve referred for the deduction, which was verified by GC-MS analysis.

### Total lignin assay

Total lignin content was measured by the two-step acid hydrolysis method according to the Laboratory Analytical Procedure of the National Renewable Energy Laboratory with minor modifications as described by Wu et al. (2013) and Li et al. (2015). The acid-insoluble lignin (AIL) was calculated gravimetrically as acid-insoluble residues after subtraction for ash, and the acid-soluble lignin (ASL) was detected by UV spectroscopy. All experiments were carried out in technological triplicate.

### Hemicellulose monosaccharide analysis by GC-MS and lignin monomer detection by HPLC

GC/MS analysis was conducted with SHIMADZU GCMSQP2010 Plus according to Xu et al. (2012). This analysis used a Restek Rxi-5 ms, 30 m × 0.25 mm ID × 0.25 um df column. Monosaccharide standards including L-rhamnose, L-arabinose, L-fucose, D-xylose, D-galactose, D-glucose, and D-mannose, were obtained from Sinopham Chemical Reagent Co., Ltd. The GC-MS Analytical Conditions are listed as the follows: Carrier gas: He. Injection Method: Split. Injection port: 250°C, Interface: 250°C. Injection Volume: 1.0 μL. The temperature program: from 170°C (held for12 min) to 220°C (held for 8 min) at 3°C/min. Ion source temperature: 200°C, ACQ Mode: SIM. The mass spectrometer was operated in the EI mode with ionization energy of 70 ev. Mass spectra were acquired with full scans based on the temperature program from 50 to 500 m/z in 0.45 s. Calibration curves of all analytes routinely yielded correlation coefficients 0.999 or better. Peaks were identified by mass profiles and/or retention times of standards. Monosaccharides were quantified based on standard curves.

Lignin monomers were determined by HPLC as previously described by Xu et al. (2012). Standard chemicals, including *p*-Hydroxybenzaldehyde (H), guaiacy (G), and syringaldehyde (S), were purchased from Sinopharm Chemical Reagent Co., Ltd. The samples after pretreatment were used to detect lignin monomers by HPLC. The solution was filtered with membrane filter (0.22 μm). The filtered solution was injected into HPLC with 20 μL (Waters 1525 HPLC) column Kromat Universil C18 (4.6 mm × 250 mm, 5 μm) operating at 28°C with CH_3_OH: H_2_O: HAc (25:74:1, v/v/v) carrier liquid (flow rate: 1.1 mL/min).

### Measurement of cellulose crystallinity

The sample preparation and cellulose crystallinity index (CrI) determination were conducted as previously described by Zhang et al. (2013). X-ray diffraction (XRD) method was used to determine cellulose crystallinity index (CrI) with Rigaku-D/MAX instrument (Uitima III, Japan). The raw biomass powder was laid on the glass sample holder (35 × 50 × 5 mm) and detected under plateau conditions. Ni-filtered Cu Kα radiation (λ = 0.154056 nm) generated at voltage of 40 kV and current of 18 mA, and scanned at speed of 0.0197°/s from 10 to 45°. The crystallinity index (CrI) was estimated using the intensity of the 200 peak (I_200_, θ = 22.5°) and the intensity at the minimum between the 200 and 110 peaks (I_am_, θ = 18.5°) as the follow: CrI = 100 (I_200_-I_am_)/I_200_ (Segal et al., 1959). I_200_ represents both crystalline and amorphous materials while I_am_ represents amorphous material. Standard error of the CrI method was detected at ±0.05–0.15 using five representative samples in triplicate.

### RNA sequencing

RNA extraction was performed as previously described by Liu et al. (2016). The raw data were cleaned by removing reads containing the poly-N or adapter and low-quality reads, which obtained from Illumina sequencing. The quality of the raw reads was assessed using FastQC (version 0.10.1) (http://www.bioinformatics.bbsrc.ac.uk/projects/fastqc/). The high quality data was used in all downstream analyses. After trimming low-quality bases (*Q* < 20) from the 5′ and 3′ ends of the remaining reads, the resulting high-quality reads were mapped onto the *Nipponbare* reference genome collected from Ensemble v17 using tophat (Sigurgeirsson et al., 2014). HTseq version 0.5.3p9 was used to convert aligned reads into counts per gene using the Ensembl v17 annotation of the rice genome (Fatima et al., 2014). The above analysis used the number of reads mapping over the entire gene length to identify differentially expressed genes. The expression level for each transcript was calculated as RPKM (Reads Per Kilobase exon Model per Million mapped reads)-derived read counts based on the number of uniquely mapped reads that overlapped with exonic regions. We used the DESeq R package (1.12.0) to identify the differentially expressed genes (DEGs) between mutant and wide-type (Burden et al., 2014). Then *P*-values were adjusted using the Benjamini and Hochberg method. A corrected *P*-value of 0.01 and log2 (fold change) of one were set as the thresholds for significant differential expression.

### Measurement of grain quality

One thousand-grain weight was converted by calculating on the weight of 200 grains. Head rice yield was calculated as the percentage of intact grains to the total number of grains after milling. The measurement of grain chalkiness rate was the percentage of chalky grains in the total number of dehulled grains. The area of chalkiness in the grain was used as the measurement of chalkiness degree (Li et al., 2014). Grain was ground to flour, which was used for measuring amylose content and gel consistency, the two parameters for eating and cooking qualities, following the methods of Wang et al. (2007).

## Results

### The *fC116* mutant has reduced mechanical strength and slightly defected growth

**Figure 1:**
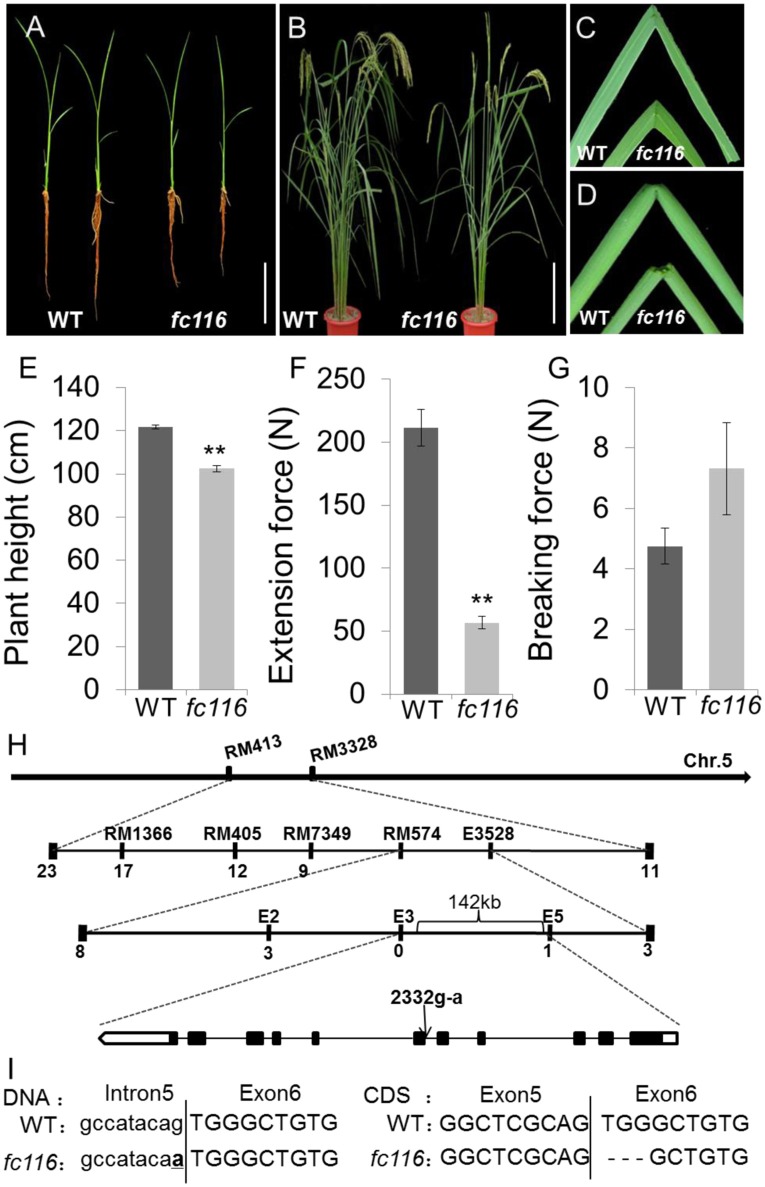
**Phenotypes and map-based cloning of the gene for *fc116*. (A)** Three-week-old wild-type and *fc116* plants. **(B)** Three-month-old wild-type and *fc116* plant. **(C,D)** The mutant leaves and internodes are easily broken. **(E)** Plant height of wild-type and *fc116*. **(F,G)** Measurement of the force required for pulling and breaking wild-type and *fcll6* mutant internodes. **(H)** The *fc116* locus was mapped to a 142 kb region on chromosome 5. Black boxes represent exons and black lines represent introns. **(I)** Sequencing analysis revealed a point mutation in genomic DNA that results 3 bp deletion in *fc116* CDS. Bar = 5 cm **(A,B)**. ^**^Significantly different (*t* = test at *P* < 0.01) compared with wild-type **(E,F)**.

The prominent phenotype of the *fc116* plants is the brittle internodes, sheaths, and leaves after booting stage (Figures 1A–D). We quantitatively measured the extension force (EF), a criterion indicating the elasticity of the plant organs, in the wild and mutant types of the rice accession. The organ with lower EF is more brittle than those with higher EF. We found that EF of the *fc116* mutant in culms was reduced by 67% comparing to its wild type at the heading stage (Figure 1F). However, the breaking force (BF), a parameter for lodging resistance, of the mutant was 36% higher than the wild type (Figure 1G). At the seedling stage, the mutant plants were not brittle, which was different from other brittle culm mutants that show brittle culms at seedling stage. The *fc116* mutant plants had slightly shorter stature/roots and fewer tillers than wild-type plants (Figures 1A,B). The mutant showed decreased plant height and tillers at the maturity stage although the growth period of the mutant was similar to the wild type (Figures 1B,E). Therefore, the mutation caused not only reduction in mechanical strength of plant parts, but also abnormalities in the growth and development. However, *fc116* showed no changes in seed weight, spikelet size, grain qualities, and lodging resistance (Table S1, Figure S1). We noticed that the mutant plants of BC14//OsNST1 encoding a UDP-glucose transporter showed additional morphological abnormalities including shortened stature and reduced seed size and fertility.

### Map-based cloning of the *FC116* locus

All F_1_ progeny derived from the both crosses *fc116*/MH63 and *fc116*/9311 showed a normal phenotype similar to that of the wild-type. Tests of heterozygotes with F_2_ progeny yielded a segregation of 6012 normal and 1917 brittle plants in *fc116*/MH63, and 7939 normal and 1842 brittle plant in *fc116*/9311 [*X*^2^ (3:1) = 0.37 and 0.31, < *p*_(0.05)_ = 4.16 and 3.98], indicating that the brittle phenotype of the *fc116* mutant was due to a recessive mutation at a single nuclear gene locus. After screening with 289 simple sequence repeat (SSR) markers that distribute evenly across the 12 rice chromosomes, the mutant locus was mapped between markers *RM413* and *RM3328* on chromosome 5 (Figure 1H). Fine-mapping further placed this locus in the 132-kb genomic region between markers *E3* and *E5*, which includes 9 predicted ORFs (Figure 1H).

**Figure 2:**
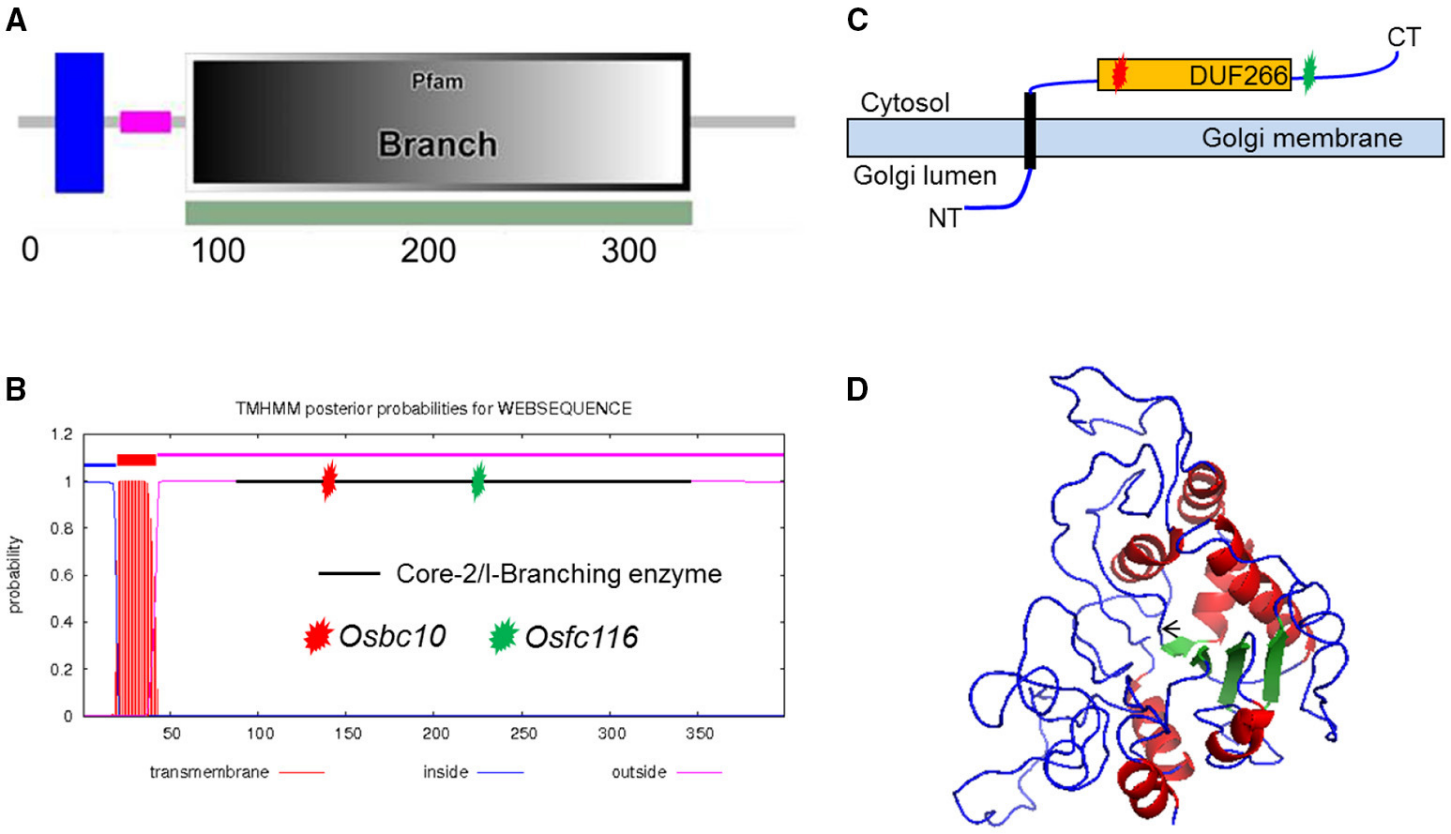
**Analysis of FC116 protein. (A,B)** Prediction of FC116 protein domain by the SMART and TMHMM2.0 program, showing a transmembrane helix domain between amino acid residues 20 and 42, a core-2/I-Branching enzyme between amino acid residues 86 and 345. Red star represents the mutation site of *bc10*; green star represents the mutation site of *fc116*. **(C)** Schematic diagram of the FC116 protein location in the Golgi membrane. **(D)** Three dimensional structure of FC116 protein. The black arrow was the deletion.

After sequencing all of the candidate genes, a point mutation was found in the junction point of the 5th intron and 6th exon of an ORF (LOC_Os05g07790), which was annotated as DNA-binding protein by the Rice Genome Annotation Project (http://rice.plantbiology.msu.edu/). The point mutation resulted in a 3 bp deletion at the beginning of the 6th exon of the coding sequence (CDS) of the mRNA of LOC_Os05g07790. That may be caused by a change in splicing pattern of the mRNA because the substitution mutation was in the recognition site for mRNA splicing (Figure 1I). This gene is allelic to *bc10*, that was reported had a mutation within the domain of a plant-specific domain with unknown function domain 266 (DUF266) located in a Core-2/I-Branching enzyme functional motif (PF02485) (Zhou et al., 2009; Figure 2A). The *fc116* mutation occurs not in DUF266 domain (Sonnhammer et al., 1988; Krogh et al., 2001) (Figures 2B,C). The predicted three dimension structure of the protein was not changed (Figure 2D).

### *FC116* encodes a putative beta-1, 6-N-acetylglucosaminyltransferase protein

**Figure 3:**
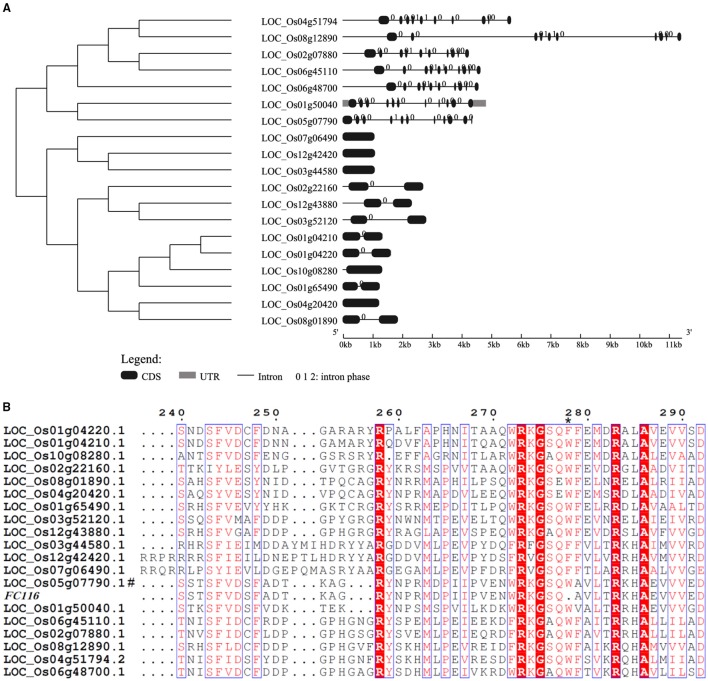
**Phylogenetic tree and protein sequences alignment of FC116 protein family. (A)** Unrooted tree of FC116 protein family and organization of exons and introns of the corresponding genes. **(B)** Black asterisk represents the deletion amino acid.

A BLASTX search for *FC116* homologs identified 19 DUF266-containing proteins in the *Oryza sativa* (*japonica*) genome (Figure 3A). This is a family of two different beta-1, 6-N-acetylglucosaminyltransferase enzymes, I-branching enzyme and core-2 branching enzyme. I-branching enzyme is responsible for the production of the blood group I-antigen during embryonic development. Core-2 branching enzyme forms crucial side-chain branches in O-glycans. An unrooted phylogenetic tree was generated from the alignment of 19 DUF266-containing protein sequences into two distinct clusters (Figure 3A). Intron-exon pattern also revealed that these homologs are clustered into two major clades, implying that they may be related but have distinct functions (Figure 3A). *FC116* is in Cluster I that have more introns than Cluster II and the closest homolog is LOC_Os01g50040. We found that the mutation occurred within a new highly conserved protein motif “R, RXG, RA” in this family (Figure 3B), The motif “R, RXG, RA” is also located in PF02485, but outside the “domain with unknown function 266” (DUF266) which has been proposed to be crucial for the protein function in a previous study (Zhou et al., 2009).

### *FC116* is ubiquitously expressed in multiple tissues and organs

**Figure 4:**
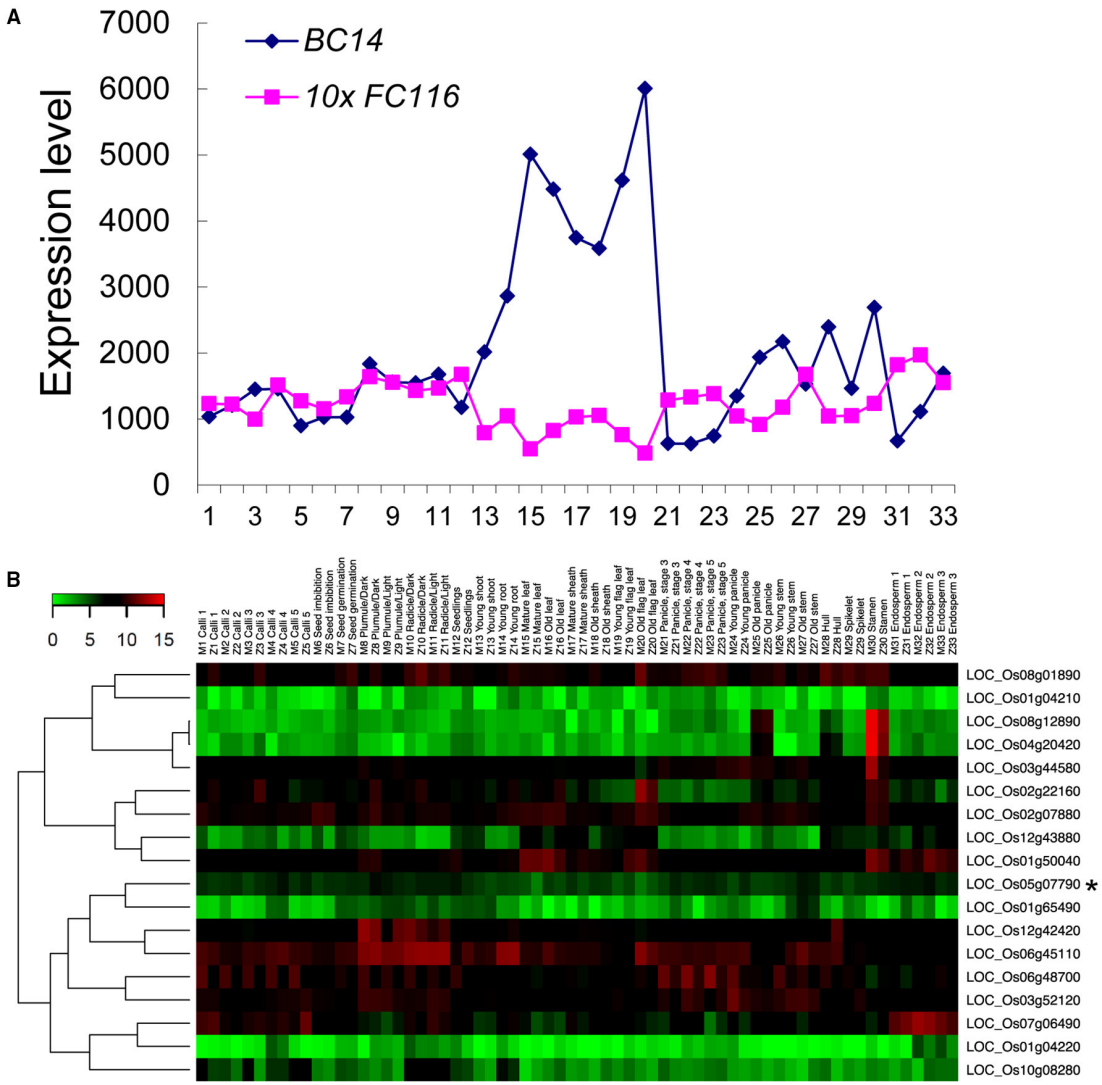
**Co-expression profiling of the *FC116* family in rice. (A)** Expression patterns of the *bc14* and *fc116* in rice. The X-axis indicates the tissues at the developmental stages with 1, Calli (15 days after subculture); 2, Calli (5 days after regeneration); 3, Calli (Screening stage); 4, Calli (15 days after induction T2); 5, Calli (15 days after induction T3); 6, Seed imbibition; 7, Seed germination; 8, Plumule (48 h after emergence, Dark); 9, Plumule (48 h after emergence, Light); 10, Radicle (48 h after emergence, Dark); 11, Radicle (48 h after emergence, Light); 12, Seedling; 13, Young shoot; 14, Young root; 15, Mature leaf; 16, Old leaf; 17, Mature sheath; 18, Old sheath; 19, Young flag leaf; 20, Old flag leaf; 21, Young panicle stages 3 (secondary branch primordium differentiation stage); 22, Young panicle stages 4 (pistil/stamen primordium differentiation stage); 23, Young panicle stages 5 (pollen-mother cell formation stage); 24, Young panicle; 25, Old panicle; 26, Young stem; 27, Old stem; 28, Hull; 29, Spikelet; 30, Stamen; 31, Endosperm (7 days after pollination); 32, Endosperm (14 days after pollination); 33, Endosperm (21 days after pollination). The Y-axis represents the expression values obtained from the microarray analysis. **(B)** *fc116* co-expression patterns in two rice varieties during the life cycles. The color scale representing the relative signal values is shown (green refers to low expression; black refers to medium expression and red refers to high expression). Black asterisk is the *fc116.*

The data for expression pattern analysis of the *FC116* gene downloaded from the CREP database (http://crep.ncpgr.cn) which was created during a rice transcriptome project using the Affymetrix Rice GeneChip microarray by rice group in our university (Wang et al., 2010a). *FC116* exhibited a ubiquitous expression in most of the 33 tissues sampled from the entire life cycle of the rice plants (Figure 4A, Figure S2). It was moderately but ubiquitously expressed in almost all tissues with relatively high levels in the endosperm, plumule, and radicles, leaf, and root at three leaf stages, stem at heading stage (Figure 4A, Figure S2). These tissues were distinct in cell wall constitutions with the variation in contents of cellulose (2.3–26.6%), hemicelluloses (2.3–32.5%), and pectins (0.9–3.3%) as we measured in previous studies (Wang et al., 2010b; Guo et al., 2014).

We further examined the expression pattern of the members in the *FC116* gene family. It was found that the absolute expression levels of the many other members in this family were much higher than *FC116*, even in stem (Table S3). Based on the hierarchical cluster analysis, this family can be classified into two major groups with ten distinct sub-groups that exhibited a complementary expression pattern in 33 tissues from the entire life cycle of two rice varieties (Figure 4B). The closest co-expressed gene was LOC_Os01g65490 that was classified into a different cluster with *FC116* according to their protein sequence similarity, while the closest homolog LOC_Os01g50040 was quite different in expression. This may indicate that the function of *FC116* is unique in this family. Thus, the loss of function in *fc116* cannot be completely compensated by other members in the family.

### Gene co-expression module analyses of *FC11*6 with cesa family and other brittle culm genes

Gene co-expression analyses can reveal functional relationships between gene products. Investigating any possible genes outside the family that are co-expressed with *FC116* may provide evidence for the functional role of FC116. We identified the top 100 positively co-expressed genes and top 100 negatively co-expressed genes with *FC116* (Table S4). We could not find highly co-expressed genes genome-wide with *FC116* as we used the Pearson's correlation coefficient (PCC) to quantify the association between two genes in the genome (r_max_ = 0.53). Go analysis indicated that some of these genes may be involved in cell wall biosynthesis such as GPI-anchored protein, fasciclin-like arabinogalactan, and wall-associated receptor kinase (Table S4). Since the gene co-expression Modules were established for rice cell wall in a previous study (Guo et al., 2007), we investigated the Modules these co-expressed gene belong to. Interestingly, we found that most negatively co-expressed genes of *FC116* belonged to Modules in close vicinity of modules 44 that typically were for secondary cell wall (SCW), whereas the positively co-expressed genes distributed to the modules including the modules 24 that typically were for primary cell wall. We also found that the expression of *FC116* was negatively correlated with the contents of cellulose and xylose (*r* = −0.4 and −0.4). These results indicate that *FC116* is not the gene that is typically for SCW although its mutation causes impaired cell wall thickening.

**Figure 5:**
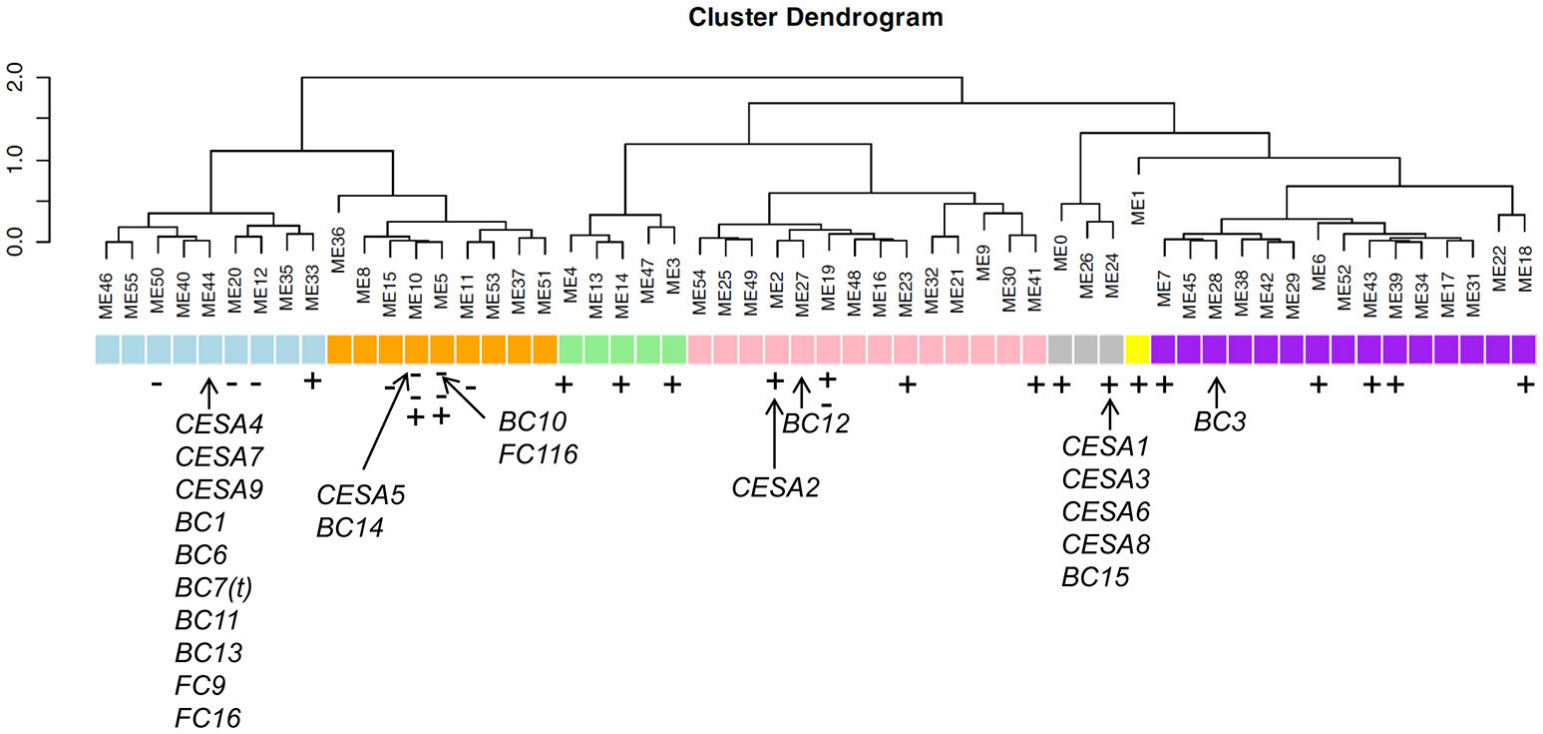
**Certain modules correlate with specific cell wall content in rice modified from Guo et al. (2014)**. Arrows represent the corresponding modules of the brittle culm (*BCs*), fragile clum (*FCs*) and *CESA* genes. Minus and plus represent the down and up regulation genes in *fc116*, respectively. Two minuses represent more down regulation genes in the module. Blue represent the modules secondary cell wall biosynthesis. Pink represent the modules of cellulose, xylose, arabinose, and guaiacyl lignin biosynthesis. Gray represents the modules of primary cell wall biosynthesis. Purple represent the modules of arabinose and galactose biosynthesis.

We also marked the *CESA* family and the brittle culm genes (*bcs)* to the modules and found that the *CESA* were distributed to the Modules 2, 10, 24, and 44, while the *BC* genes were distributed to the Modules 5, 10, 24, 27, 28, and 44 (Figure 5; Table S5). Strikingly, the genes contained in Module 44 included those for cellulose synthases *CESA4, CESA7, CESA9, BC1 (OsCOBRA), BC6 (CESA9)* (Kotake et al., 2011), *BC7(t) (CEAS4), BC11 (CESA4), BC13 (CESA9)* (Song et al., 2013), *FC9 (OsMYB103)*, and *FC16 (CESA9)*, while the Module 24 included the *CESA1, CESA3, CESA6, CESA8*, and *BC15/OsCTL1* (chitinase-like). FC116/BC10 belongs to Modules 5 that in close vicinity of Modules 10 contain the *CESA5* and *BC14* (Figure 5). Interestingly, we found significant negative co-expression of *FC116/BC10* with *BC14* (*r* = −0.41). We noticed that *FC116*/*BC10* and *BC14* showed overlapped expression at the seedling stage; however, they showed divergence in expression tendency in many tissues that underwent secondary growth from the booting stage (Figure 4A). It is indicate that the causing of the brittle/fragile culms can be complicated and the overlapping or the close vicinity of these Modules imply their relationship that worth further investigation.

### *fc116* has altered cell wall structure and composition

**Figure 6:**
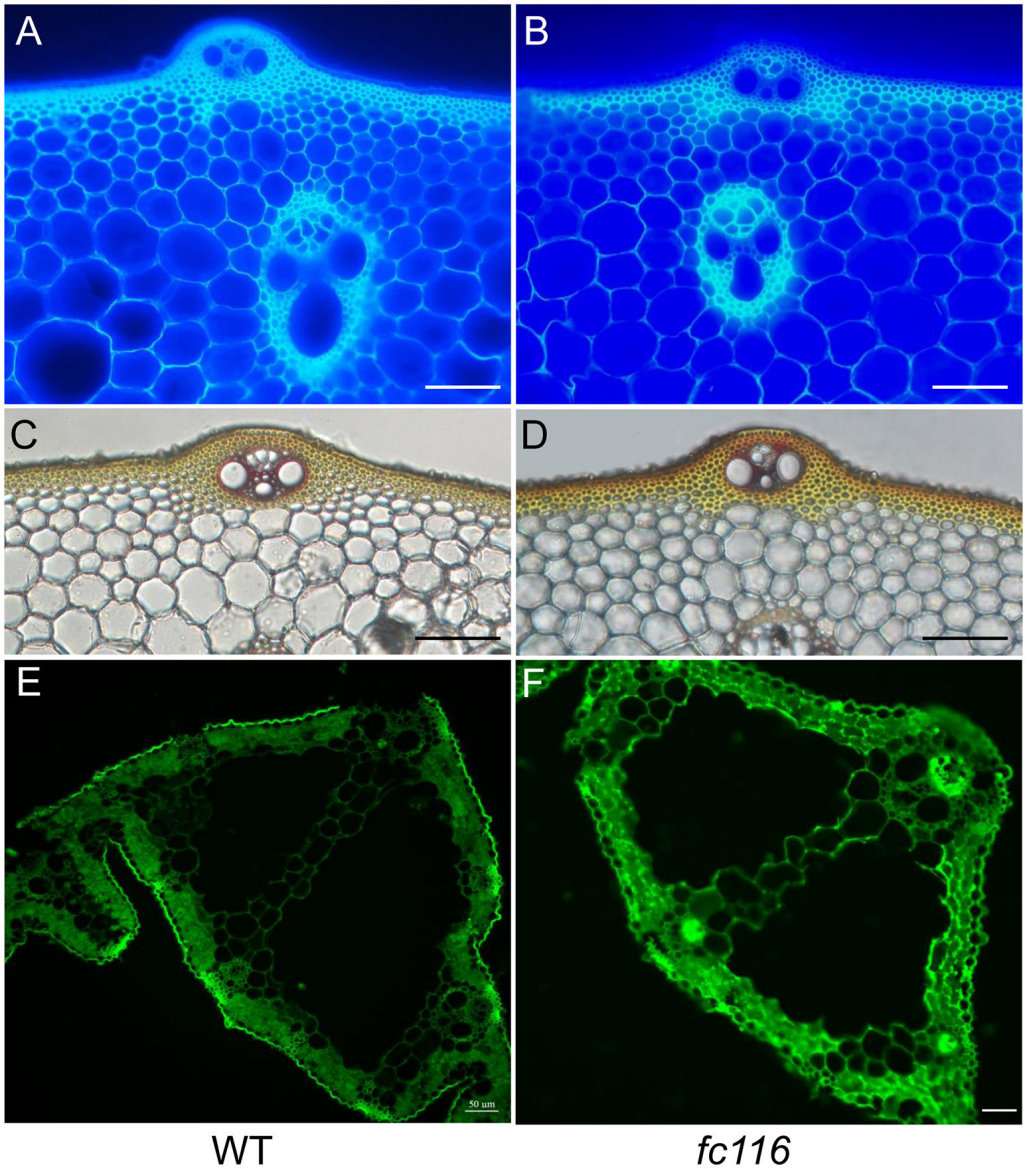
**Staining of cellulose, lignin, and hemicellulose in wild-type and *fc116* plants. (A)** and **(B)** Calcofluor staining of the transverse culm sections of wild-type **(A)** and *fc116* **(B)** plants, showing the decreased level of cellulose in the cell walls in the mutant culm. **(C,D)** Wiesner's staining of the transverse culm sections of wild-type **(C)** and *fc116* **(D)**, showing the increased level of lignin in the walls of sclerenchyma cells. **(E,F)** Immunohistochemical localization of xylan in leaf sections of wild-type and *fc116* plants, showing the increased fluorescent signals in all cells visualized with xylan antibody CCRC-M148. Bar = 50 μm.

In rice internodes, peripheral vascular tissue cells and sclerenchyma cells under the epidermal layer develop thickened cell walls, which are presumed to provide mechanical strength to the plants. To determine the alterations in cell wall structure and thickness that are responsible for the brittle phenotype, we compared the cell wall morphology in the internodes between *fc116* and its wild type by microscopy. Cellulose and other cell wall components showed fluorescence staining with calcofluor, while phloroglucinol reacted with coniferaldehyde groups in lignin and the color intensity approximately reflects the total lignin content. In transection, the peripheral sclerenchyma in wild-type internodes showed substantial staining with calcofluor white, while *fc116* internodes were slightly stained, and have an apparent defect in cell wall thickening (Figures 6A,B). The phloroglucinol staining of the transection indicated increased lignin content in the peripheral sclerenchyma and vascular bundles in *fc116* and again decreased cell wall thickening compared with the wide type (Figures 6C,D). We further observed that *fc116* generated stronger fluorescent signals in the vascular bundle cells than in wild-type plants using CCRC-M148 antibody recognizing xylan (Figures 6E,F). These results suggest that the *fc116* mutation affects the formation of the secondary cell walls, particularly in the sclerenchyma tissues.

The culms of *fc116* mutant plants showed 45% reduction in cellulose content and 34% increase in hemicellulose and 18% in lignin (Table 1). In addition, *fc116* exhibited lower crystallinity index (CrI) than the wild type in both crude cell wall fraction and crude cellulose. However, the CrI changes in crude cellulose were not as significant as in the crude cell wall fraction. This was different from the mutant *fc16*/*OsCESA9* (another mutant maintained in our lab) that showed significant decrease in CrI in crude cellulose but slightly decrease in CrI of the crude cell wall fraction (data not shown). The monosaccharide composition analysis showed that the amount of pentoses (xylose and arabinose) in the hemicelluloses was significantly increased in the mutant, while the all hexose (Glucose, Mannose, Galactose) were decreased. Compared with the wild type, *fc116* had 10 and 14% increase of Ara and Xyl, respectively (Table 2). We also found the ratio of Ara/Xyl significantly decreased despite of increasing absolute value of Ara. Similar trend in the change of polysaccharides was also found with the mutant in the pectic fraction (Table 1). The changes in the cell wall content were consistent with the staining analysis. Our results were basically consistent with the previous study that reported significant decrease in cellulose and increase in lignin in *bc10* (Zhou et al., 2009). Our results imply that both cellulosic and non-cellulosic polysaccharide synthesis is widely impaired in *fc116*. We noticed that the *OsCESA4* mutant of *Nipponbare*, displayed 60% reduction in cellulose content and a twofold compensatory increase in arabinoxylan (Zhang et al., 2009). BC15 (chitinase-like) which was predicated to have binding sites to N-acetylglucosamine also showed that Ara and Xyl contents were increased by 58 and 77%, respectively by mutation despite the mutants did not show any changes in lignin content (Wu et al., 2012). The polyphenolic molecule lignin is an amorphous polymer of phenylpropane units with three monomers: *p*-hydroxyphenyl (H), syringyl (S), and guaiacyl (G). It was found that the contents of the all three monomers H, S and G in the mutant plants were significantly increased compared to the wider type. However, the relative amount of H monomer (% of the total monomers) slightly decreased, whereas the S and G% slightly increased (Table 3). These results suggest that *fc116* mutation might cause equal increase of the every individual steps, but under such circumstances, the efficient of the branch leading to three different major monomers can be slightly different as they share and thus compete for limited precursors.

**Table 1:** **Comparison of cell wall components between wild-type and *fc116* culms**.

**Cell wall fraction**		**WT**	***fc116***
Pectins	Hexose	1.47 ± 0.17	0.88 ± 0.13^*^
(%)	Pentose	0.31 ± 0.03	0.35 ± 0.02
	Uronic acid	0.40 ± 0.01	0.50 ± 0.03^*^
	Total	2.18 ± 0.19	1.73 ± 0.15^**^
Hemicelluloses	Hexose	1.41 ± 0.05	1.40 ±0.19
(%)	Pentose	21.02 ±0.35	25.55 ± 0.45^**^
	Total	22.43 ± 0.39	26.89 ± 0.48^**^
Cellulose	Content	30.56 ± 0.48	25.12 ± 0.65^**^
(%)	CrI 1	53.75	46.11^*^
	CrI 2	44.68	43.32
Lignin	ASL	4.13 ± 0.05	4.07 ±0.07
(%)	AIL	10.17 ± 0.28	12.77 ±0.14^**^
	Total	14.30 ± 0.29	16.84 ± 0.18^**^

* and ** Significantly different (t-test at P < 0.05 and 0.01) compared with wild-type (n = 3) ± SD. Each wall component was calculated as percentage of dry matter. CrI 1, crystallinity index of crude cell wall; CrI 2, crystallinity index of crude cellulose; ASL, acid soluble lignin; AIL, acid-insoluble lignin.

**Table 2:** **Monosaccharide composition of hemicelluloses in *fc116* compared to wild-type**.

	**4N KOH-extractable**		**Non-KOH-extractable**		**Total**	
	**WT**	***fc116***	**WT**	***fc116***	**WT**	***fc116***
Rhamnose	0.16 ± 0.03	0.15 ± 0.06	0.13 ± 0.02	0.13 ± 0.00	0.29 ± 0.03	0.28 ± 0.06
Fucose	ND	ND	0.01 ± 0.00	0.01 ± 0.00	0.01 ± 0.00	0.01 ± 0.00
Arabinose	4.07 ± 0.27	4.44 ± 0.65	2.14 ± 0.27	2.39 ± 0.23	6.21 ± 0.28	6.83 ± 0.54^*^
Xylose	50.60 ± 1.23	59.94 ± 1.57^**^	12.95 ± 1.54	12.31 ± 0.73	63.55 ± 1.56	72.25 ± 1.56^**^
Mannose	0.03 ± 0.02	0.03 ± 0.01	0.05 ± 0.00	0.03 ± 0.00	0.08 ± 0.01	0.06 ± 0.00
Glucose	1.28 ± 0.05	1.15 ± 0.14	22.89 ± 2.44	9.38 ± 1.05^**^	24.17 ± 2.45	10.53 ± 1.02^**^
Galactose	0.68 ± 0.02	0.65 ± 0.07	1.42 ± 0.34	1.11 ± 0.04	2.10 ± 0.03	1.76 ± 0.06
Ara/Xyl	0.08	0.07	0.17	0.19	0.10	0.09

* and ** Significantly different (t-test at P < 0.05 and 0.01) compared with wild-type (n = 3) ± SD. Each monosaccharide component was calculated as mg g^−1^ of dry matter. ND, none detected.

**Table 3:** **Lignin monomer composition of the *fc116* compared to wild-type**.

	**H**	**G**	**S**	**total**	**H/G**	**S/G**	**S/H**
WT	78.78 (35.0%)^a^	84.51 (37.5%)	62.03 (27.5%)	225.32	0.95	0.73	0.78
*fc116*	83.89^*^ (33.6%)	94.39^*^(37.7%)	71.80^*^(28.7%)	250.08^**^	0.89	0.76	0.86

* and ** Significantly different (t-test at P < 0.05 and 0.01) compared with wild-type (n = 3) ± SD. Each monomer component was calculated as μ mol g^−1^ of dry matter.

H, p-hydroxyphenyl lignin; G, guaiacyl lignin; S, syringyl lignin^a^, percentage of total monomers.

### Comparison of genome-wide expression profiles of *fC116* and wild-type

**Figure 7:**
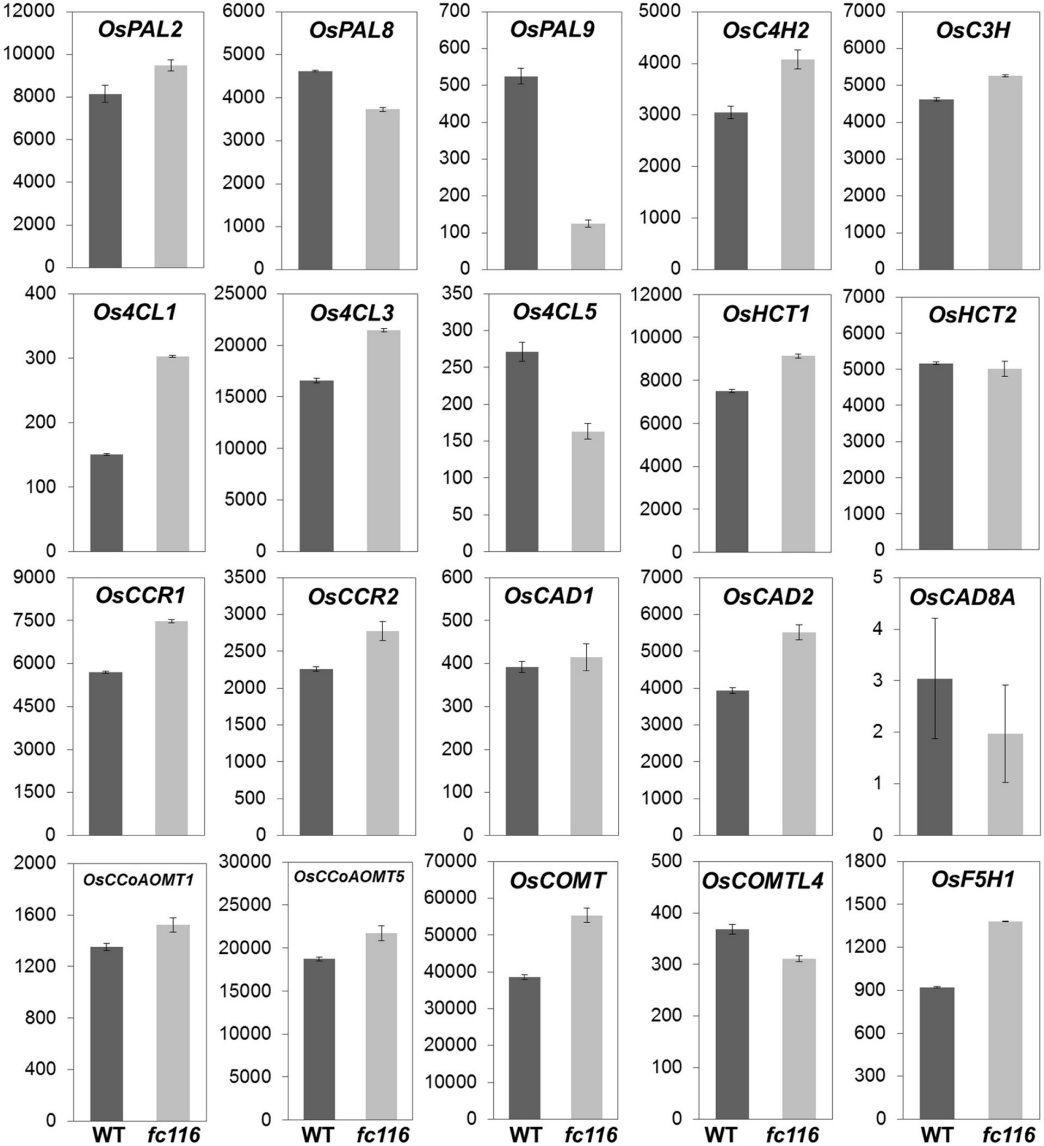
**Expression of lignin monomers biosynthesis genes between wild-type and *fc116***. The y-axis represents the raw expression values obtained from the RNA-seq analysis. C3H, p-coumarate 3-hydroxylase; C4H, trans-cinnamate 4-hydroxylase; 4CL, 4-Coumarate-CoA ligase; CAD, Cinnamyl alcohol dehydrogenase; CCoAOMT, caffeoyl-CoA 3-O-methyltransferase; CCR, cinnamoyl-CoA reductase; COMT, caffeic acid O-methyltransferase; COMTL, caffeic acid O-methyltransferase like; F5H, ferulate 5-hydroxylase; HCT, hydroxycinnamoyltransferase; PAL, phe ammonialyase.

Since the dramatical and universal changes in cell wall composition were obseved, in *fc116* plants, we detected the transcription alterations of the major genes associated with wall polymers using RNA-seq which reliably quantifies the expression levels of individual genes in multigene families. Relative to the wild type, the mutants exhibit pathway-wide up-regulation of lignin biosynthesis genes, including the 4-coumarate-CoA ligase (4CL), cinnamyl alcohol dehydrogenase (CAD), ferulate 5-hydroxylase (F5H), and cinnamoyl-CoA reductase (CCR), that catalyze specific branches of lignin biosynthesis leading to different lignin monomers, suggested possible alterations in lignin composition along with overall increase in response to FC116 mutation (Figure 7). Of the total 27 examined lignin units biosynthesis genes that showed high transcription levels in the culm at heading stage based on microarray data, 20 were identified with the expression levels beyond 200 in this RNA-seq and 18 with much higher expression levels in *fc116* than in wild-type. The similar up-regulation tendency was found in typical SCW-related Modules 40 (19 up and 5 down) and 44 (58 up and 4 down), the modules for cellulose, xylan, H, S, and G. With regard to the increased hemicellulose, Xyl and Ara levels, the mutants were detected with higher transcription levels in all three glycosyltransferase genes LOC_Os03g08600 (GT8), LOC_Os02g22380 (GT61), LOC_Os06g47340 (GT43), which proposed to be participated in hemicellulose synthesis. LOC_Os06g47340 (OsIRX14), orthologs to the putative β-1,4-xylan backbone elongating Arabidopsis IRX14 gene, was demonstrated as one of three important genes involved in backbone chain synthesis of hemicelluloses (Scheller and Ulvskov, 2010; Chiniquy et al., 2013). The only enzymes characterized so far were belong to GT8 and GT61 that decorate the xylan backbone, as the members of the GT8 family (GUX1 and GUX2) are required for glucuronosyl substitution in Arabidopsis stem (Mortimer et al., 2010), while the members from GT61 family (xylan arabinosyltransferases, XATs) responsible for α-(1,3)-arabinosyl substitution of xylan (Anders et al., 2012). The data in expression of these genes were consistent with the extensive increase in the levels of hemicelluloses and lignins. Interestingly, that only *CESA 4, 7, 9* that especially during SCW formation expression were slightly increased while the other *CESAs* remained unchanged or slightly decreased. It appears to differ from the fact that the *CESA* genes were all co-upregulated during culm development in the normal condition and even in the *bc15* despite the dramatic decrease of the cellulose content in this mutant (Wang et al., 2010b; Wu et al., 2012). So, we conceive that in *fc116* the pathway relative typical for SCW might be activated through an unknown mechanism. It was observed that the BC12/GDD1 (kinesin-like protein) can act as a transcriptional activator that might regulate KO2 gene expression in GA biosynthesis pathway, besides binding to MTs in an ATP-dependent manner (Li et al., 2011). *fc116* differ from other rice brittle culm mutants such as *bc1* and *bc15* that show no obviously compensatory changes in lignin content and the expression profile of the relevant genes (Li et al., 2003; Wu et al., 2012). It is possible that there might be multiple knobs and switches that can be tuned to execute specific regulation of different cell wall pathways in order to optimize secondary cell wall composition.

## Discussion

Plant cell walls are complex and dynamic structures composed mostly polysaccharides, highly glycosylated proteins, and lignin. In recent years, studies on the brittle culm mutants have identified several genes responsible for secondary cell wall formation, revealing many processes of monocot cell wall biosynthesis. Here, we report the isolation of a fragile culm gene *fc116*, allelic to *bc10*, which has sequence similarity with the domain to a core 2 beta-1,6-N-acetylglucosaminyltransferase (C2GnT). We investigated the changes in cell wall features, monosaccharide composition, phenolic monomers constitution, and the transcriptomic of the *fc116*. Our results are basically consistent with the previous study by Zhou et al. (2009), but have some interesting findings including: 1) *fc116* mutation occurred within a newly identified protein motif “R, RXG, RA” located outside the “domain of unknown function 266” (DUF266) which was proposed to be crucial for the function of protein BC10 (Zhou et al., 2009); 2) The three major cell wall polymers of the mutant were all significantly changed in their content, constitution and features. Also, we observed contrary changes in pentoses (Xyl and Ara) and hexoses (Glc, Man, and Gal) in hemicellulosic polysaccharides. All these findings are of interesting for further biochemical and functional characterization of protein FC116; 3) The transcriptomic profiling of *fc116* revealed the genetic regulation of the content and monomers of hemicelluloses and lignins.

### *FC116* was neither a gene for typical secondary cell wall nor for typical primary cell wall biosynthesis

The FC116/BC10 gene was lowly but ubiquitously expressed in many tissues and organs that showed distinct cell wall constitutions. It was expressed in the all tissues examined across the whole life cycle of rice, and slightly down-regulated during secondary growth. Co-expression analysis further indicated it belongs to the co-expression Modules 5 that in close vicinity of the Modules 10 containing the *CESA5* and *BC14*, without obvious inclination to gene modules for typical PCW and SCW. In addition, the expression of gene *FC116* was not regulated by *OsMYB103*, the transcription factor that typically regulates secondary cell wall biosynthesis in rice (Figure S3). We noticed that, other than *FC116*, many *BC* genes (*BC3, BC12, BC14*) also could distribute to the Modules that not typically involved in the biosynthesis of neither SCW nor PCW in spite of their prominent effects on the secondary cell wall (Figure 5). We noticed that *BC3, BC12*, and *BC14* function in the membrane vesiculation, cortical microtubule-dependent cellulose deposition and nucleotide sugar transportation, respectively (Xiong et al., 2010; Zhang et al., 2010, 2011; Li et al., 2011). These biological processes are fundamental and may be common required for both PCW and SCW formation. Although the brittle culm mutants identified in rice have commonly reduced mechanical strength caused by decreased cellulose content and altered cell wall structure, the mechanism underlying that trait could be quite complex.

### The mechanism of decreased hexose levels in cell wall fractions of mutant *fc116*

Our results revealed that *fc116* caused a universal effect on the hexoses in the cellulosic and non-cellulosic (pectins and hemicelluloses) fractions of cell wall residues. That the dramatic decrease in crystallinity of raw material other than the crude cellulose also indicated the changes of non-cellulosic fraction. Compared to its allelic mutant *bc10, fc116* exhibited significant increase in the hemicellulose content and alteration in its monosaccharide components (increased in Xyl and Ara, but decreased in Glc, Man and Gal) with up-regulated expression of genes for hemicelluloses and lignin. However, the soluble hexoses derived from matrix polysaccharides were much higher in *fc116* plants compared to wild-type, indicating the source of the sugar for cell wall biosynthesis in this fragile culm mutant was not affected (Figure S4). Our RNA-seq data further indicated that the expression levels of various genes directly involved in cellulose biosynthesis (such as CESA- and BC-encoding genes) are not impaired in *fc116* (Table S6). Thus, the alteration of the hexose polysaccharides might lie in the availability, transporting or/and delivery of the substrate for the cell wall polysaccharides biosynthesis as many studies have revealed the importance of substrate availability in polysaccharide biosynthesis (Zablackis et al., 1996; Bonin et al., 1997; Usadel et al., 2004; Diet et al., 2006; Oka et al., 2007). We noticed that the mutation of BC15/OsCTL1, a class II chitinase that may use GlcNAc-containing glycoproteins or chitooligosaccharides as the substrates, also causes universal effects on the hexoses in cellulosic and non-cellulosic polysaccharides (De Jong et al., 1993; Domon et al., 2000; Passarinho et al., 2001; Zhong et al., 2002; Hermans et al., 2010). *BC15* belongs to Module 24 typically for PCW without an obvious effect on lignin biosynthesis, but has an overlap expression pattern with the *FC116*. The mutation in BC14/OsNST1, a Golgi-localized nucleotide sugar transporter generally work together with themselves or with glycosyltransferases (GTs), also causes a decrease in the glucose content not only in cellulose but also other glucoconjugated polymers. The mechanism for universal effects of FC116 on several kinds of hexoses in both cellulosic and non-cellulosic fractions remains to be answered. Some GTs are reported displaying not strict regiospecificities and substrate specificity (Thibodeaux et al., 2008; Gantt et al., 2011; Wang et al., 2012), as well as AtUTr1 (UDP-galactose transporter) and BC14/OsNST1 (UDP-glucose transporter) have been identified to have both UDP-galactose and UDP-glucose transport activity (Norambuena et al., 2002; Zhang et al., 2011). More studies are needed to further characterize the possible molecular mechanisms of FC116 protein function in plant cell wall formation process and regulation. First, the *fc116* mutants could be characterized using more advanced tools such as immunological approaches like Glycome Profiling or ComPP (Sørensen and Willats, 2011; Pattathil et al., 2012), which would give more insights in to the glycome modification resulting from *fc116* mutation. Secondary, the FC116 could be expressed and purified to validate the actual biochemical function of this protein. Overall, our results reported here can give a hint for the further characterization of biochemical function of the FC116 protein and its crucial roles in cell-wall formation.

## Author contributions

MZ completed major experiments. FW and ZH screened the mutants and participated the genetic mapping of the *FC116*. MZ and KG analyzed the data. YL completed the immunofluorescence detection. GX, XC, and YW contributed in revision of the manuscript. LP and LW designed project, supervised experiments and finalized manuscript.

### Conflict of interest statement

The authors declare that the research was conducted in the absence of any commercial or financial relationships that could be construed as a potential conflict of interest.
