# Supplementary material for: A Novel FC116/BC10 Mutation Distinctively Causes Alteration in the Expression of the Genes for Cell Wall Polymer Synthesis in Rice
Source: Front Plant Sci. 2016 Sep 21;7:1366. doi: 10.3389/fpls.2016.01366 (PMC5030303; doi:10.3389/fpls.2016.01366)
Supplement: Supplementary file 1 [file Table1.PDF]

Table S1. Agronomic traits and processing qualities of the wild-type and *fc116*.

**Traits**		**WT**	***fc116***	
Agronomic traits				
	Plant height (cm)	121.78±3.80	102.39±1.36**	-15% [a]
	Tillers/plant	22.00±3.22	18.00±4.33**	-18%
	Dry straw (g/plant)	37.12±3.23	30.59±5.72**	-19%
	1,000-Grain weight (g)	27.35±0.25	26.81±0.22	-2%
	Lodging index	68.32±8.96	52.17±6.84**	-22%
Processing qualities				
Appearance and milling qualities				
	Grain (length/width)	1.70	1.70	0%
	Brown rice rate (%)	0.84	0.87	4%
	Head milled rate (%)	0.68	0.71	4%
	Chalky grain rate (%)	29.70	25.20	-15%
	Chalkiness degree (%)	11.70	9.50	-19%
Eating and cooking qualities				
	Amylose content (%)	17.20±0.37	18.4±0.24	7%
	Gel consistency (mm)	76.33±0.47	81.33±2.49	7%

** indicated significant difference between the wild type and *fc116* by *t*–test at $P < 0.01$.

[a] Percentage of the increased and decreased level between the wild-type and *fc116* mutant.
