# Supplementary figures and images for: A Novel FC116/BC10 Mutation Distinctively Causes Alteration in the Expression of the Genes for Cell Wall Polymer Synthesis in Rice
Source: Front Plant Sci. 2016 Sep 21;7:1366. doi: 10.3389/fpls.2016.01366 (PMC5030303; doi:10.3389/fpls.2016.01366)

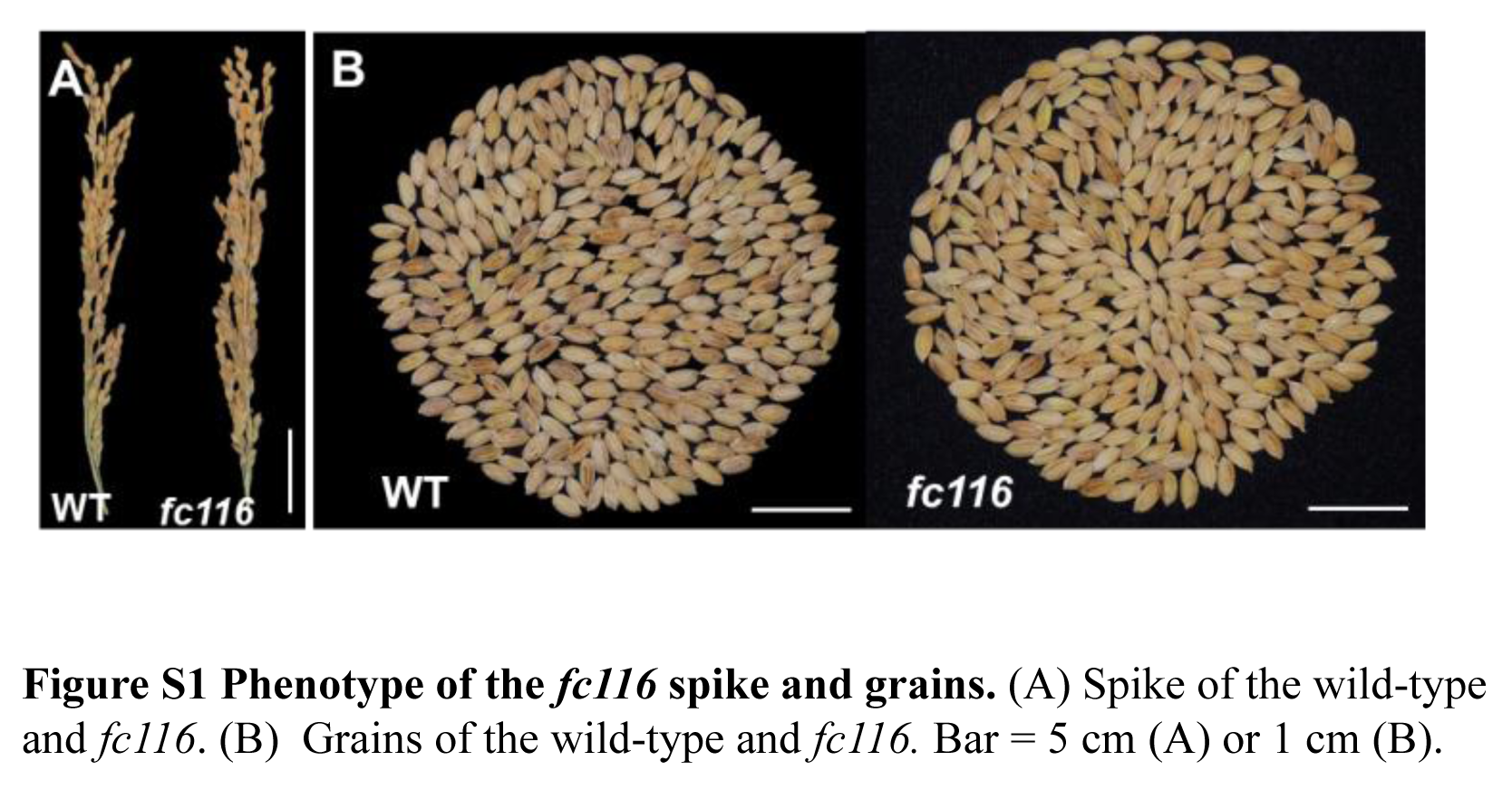

Supplement: Supplementary file 7 [file Image1.TIF]

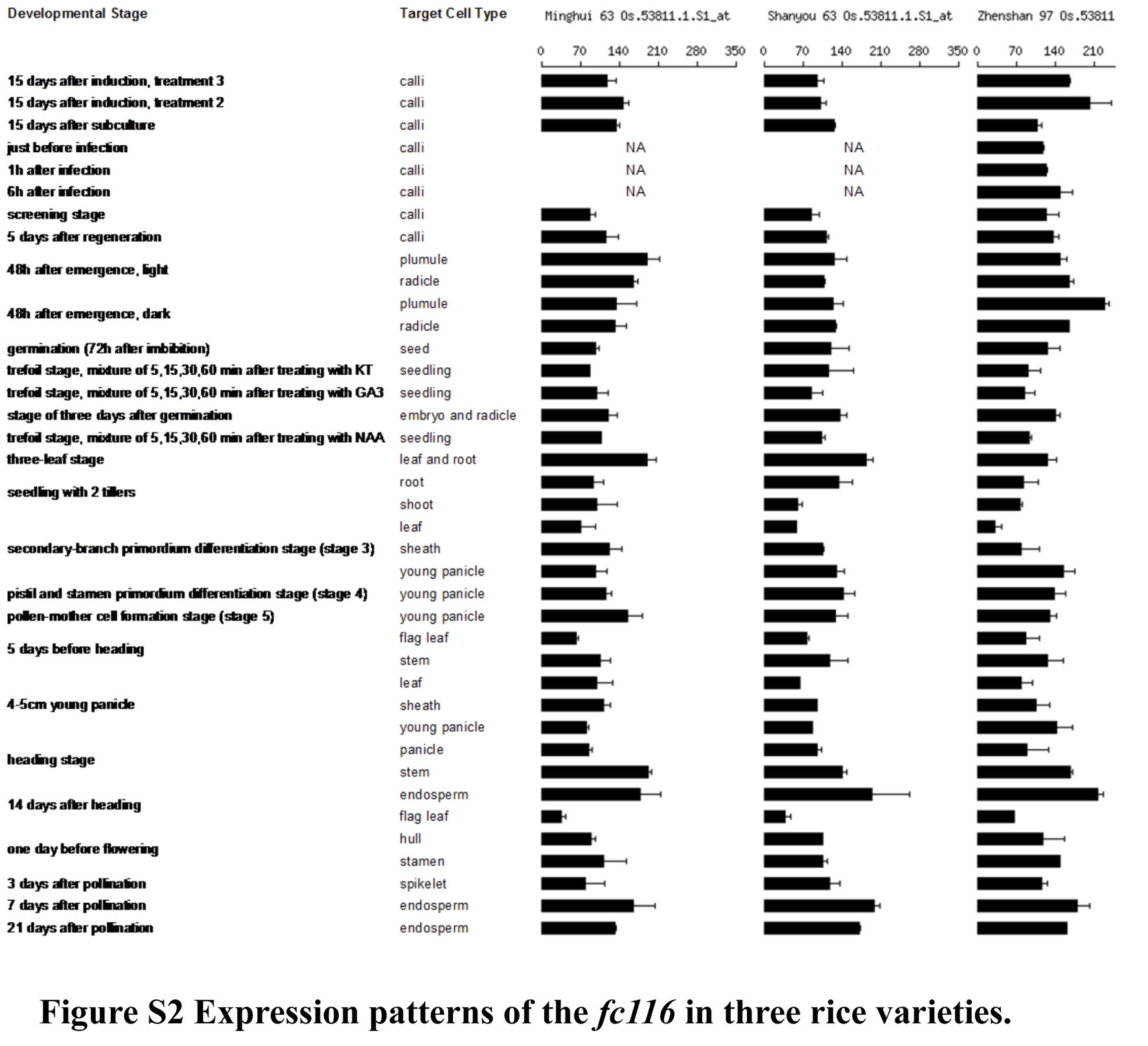

Supplement: Supplementary file 8 [file Image2.TIF]

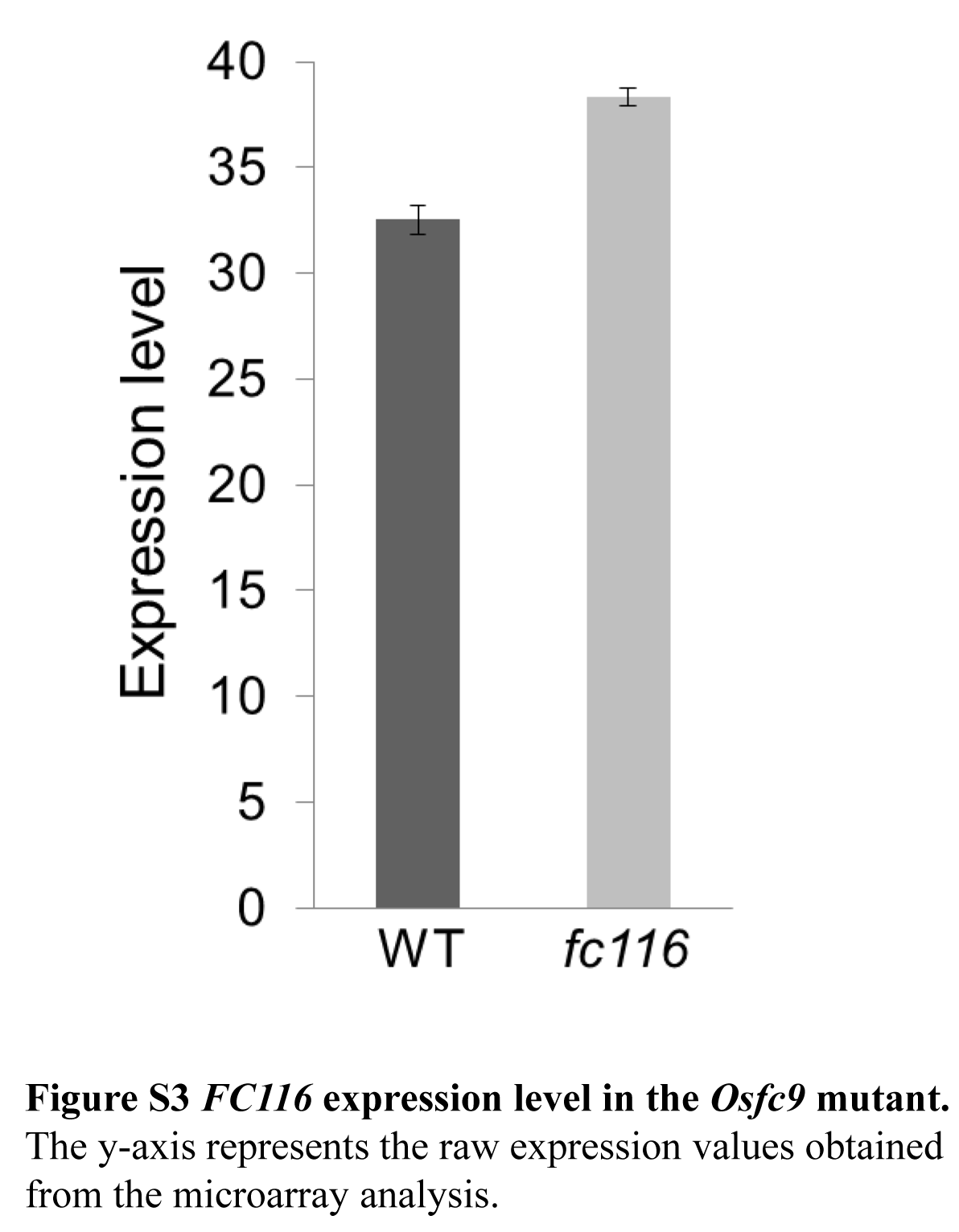

Supplement: Supplementary file 9 [file Image3.TIF]

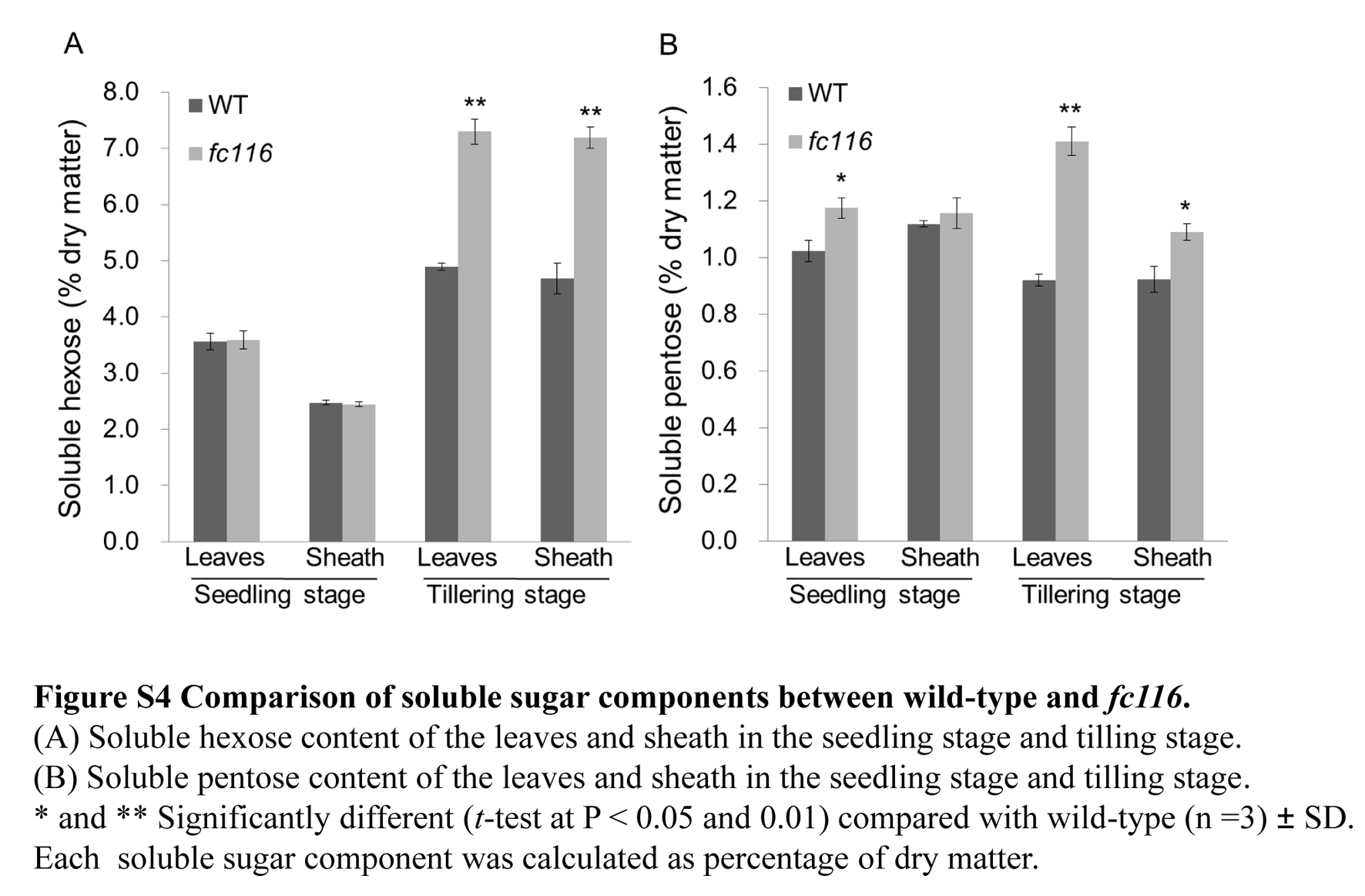

Supplement: Supplementary file 10 [file Image4.TIF]
